# The Ninth International Medical Congress

**Published:** 1886-06

**Authors:** 


### THE NINTH INTERNATIONAL MEDICAL CONGRESS.

At the meeting in St. Louis, on May 3rd, of the executive committee of the International Medical Congress of 1887, the existing vacancies in the presidencies of the congress and of sections were filled. The section of otology and laryngology was divided, and the section of laryngology was restored. Professor N. S. Davis, of Chicago, Illinois, was elected president of the congress. Supervising surgeon-general J. B. Hamilton, of the Marine Hospital service, was elected secretary-general. Professor A. B. Palmer, of the University of Michigan, was elected president of the section of pathology. Professor J. F. Harrison, of the University of Virginia, was elected president of the section of gynæcology, and Professor J. H. Callender, of the Nashville and Vanderbilt University, president of the section of physiology. Dr. H. O. Marcy requested to be transferred from the section of collective investigation, etc., to the section of gynæcology, which was done, and Medical Director A. L. Gihon, of the United States Navy, was elected president of the section of collective investigation, etc. Professor A. W. Calhoun requested transfer from the presidency of the section of ophthalmology, which was acceded to, and Professor E. Williams, of the Miami Medical College of Cincinnati, was elected president of the section of ophthalmology. Dr. W. H. Daly, of Pittsburg, Pennsylvania, was elected president of the section of laryngology.

The office of associate secretary-general was abolished.

Very satisfactory reports were made to the committee of replies received from prominent members of the medical profession in Europe, indicating their intention to attend the congress and to furnish papers.

Having completed the preliminary organization of the congress, so far as the more important official positions are concerned, and arranged the details of the work in preparation, the executive committee adjourned.

*The Enlarged Committee of Arrangements of the American Medical Association* was instructed at the meeting in New Orleans, last year, to revise the report of the preliminary work of organizing the Ninth International Congress made at that meeting, and it reported at St. Louis that it had revised the work and made some few changes in the organization, and adopted rules for the government of the Congress, and selected general officers for the congress and presidents of sections who together should constitute an *executive committee*, and that to this executive committee the affairs of the congress had been committed. This report of the committee of arrangements was received by the American Medical Association and unanimously adopted, thereby discharging its committee of arrangements and ending all official connection of the Association with the congress. Therefore the future work of preparation for the opening of the congress in September, 1887, is left entirely in the hands of the executive committee, and the local committee of arrangements in Washington.

A correct list of the officers of the congress to this date is as follows:

GENERAL OFFICERS OF THE INTERNATIONAL CONGRESS OF 1887.

*President*—N. S. Davis, M. D., LL. D., Chicago, Illinois.

*Vice-Presidents*—Wm. Brodie, M. D., Detroit, Michigan; W. W. Dawson, M. D., Cincinnati, Ohio; J. A. Grant, M. D., Ottawa, Canada; E. M. Moore, M. D., Rochester, New York; Tobias G. Richardson, M. D., New Orleans, Louisiana; Lewis A. Sayre, M. D., New York, N. Y.; J. M. Toner, M. D., Washington, D. C.; The President of the American Medical Association; The Surgeon-General of the United States Army; The Surgeon-General of the United States Navy; The Supervising Surgeon-General of the United States Marine Hospital Service.

*Secretary-General*—John B. Hamilton, M. D., Washington, D. C.

*Treasurer*—E. S. F. Arnold, M. D., F. R. C. S., New York, N. Y.

*Chairman of the Finance Committee*—Richard J. Dunglison, M. D., Philadelphia, Pennsylvania.

*Executive Committee of the Congress*—Henry H. Smith, M. D., Chairman, Philadelphia, Pennsylvania; Abram B. Arnold, M. D., Baltimore, Maryland; E. S. F. Arnold, M. D., F. R. C. S., New York, N. Y.; Wm. T. Briggs, M. D., Nashville, Tennessee; J. H. Callender, M. D., Nashville, Tennessee; N. S. Davis, M. D., LL. D., Chicago, Illinois; F. S. Dennis, M. D., F. R. C. S., New York, N. Y.; Richard J. Dunglison, M. D., Philadelphia, Pennsylvania; Albert L. Gihon, M. D., Medical Director U. S. Navy, Washington, D. C.; John P. Gray, M. D., LL. D., Utica, New York; John B. Hamilton, M. D., Washington, D. C.; James F. Harrison, M. D., Virginia; S. J. Jones, M. D., LL. D., Chicago, Illinois; Joseph Jones, M. D., New Orleans, Louisiana; DeLaskie Miller, M. D., Chicago, Illinois; A. B. Palmer, M. D., LL. D., Ann Arbor

Michigan; Wm. H. Pancoast, M. D., Philadelphia, Pennsylvania; A. R. Robinson, M. D., New York, N. Y.; J. Lewis Smith, M. D., New York, N. Y.; Jonathan Taft, M. D., Cincinnati, Ohio; F. H. Terrill, M. D., San Francisco, California; E. Williams, M. D., Cincinnati, Ohio.

*Local Committee of Reception and Arrangements*—A. Y. P. Garnett, M. D., Washington, D. C., Chairman; The Surgeon-General of the U. S. Army; The Surgeon-General of the U. S. Navy; The Supervising Surgeon-General of the U. S. Marine Hospital Service; J. H. Baxter, M. D., U. S. Army; C. H. A. Kleinschmidt, M. D., Washington, D. C.; N. S. Lincoln, M. D., Washington, D. C., J. M. Toner, M. D., Washington, D. C.

PRESIDENTS OF THE SECTIONS.

*General Medicine*—Abram B. Arnold, M. D., Professor of Clinical Medicine, Baltimore, Maryland.

*General Surgery*—William T. Briggs, M. D., Professor of Surgery, Nashville, Tennessee.

*Military and Naval Medicine and Surgery*—Henry H. Smith, M. D., formerly Professor of Surgery and Surgeon-General of Pennsylvania, Philadelphia, Pennsylvania.

*Obstetrics*—DeLaskie Miller, M. D., Ph. D., Professor of Obstetrics, Chicago, Illinois.

*Gynæcology*—James F. Harrison, M. D., Professor of Obstetrics, etc., University of Virginia.

*Therapeutics and Materia-medica*—F. H. Terrill, M.D., Professor of Therapeutics, San Francisco, California.

*Anatomy*—William H. Pancoast, M. D., Professor of Anatomy, Philadelphia, Pennsylvania.

*Physiolgy*—J. H. Callender, M. D., Professor of Physiology, Nashville, Tennessee.

*Pathology*—A. B. Palmer, M. D., LL. D., Professor of Pathology, etc., Ann Arbor, Michigan.

*Diseases of Children*—J. Lewis Smith, M. D., Professor of Diseases of Children, New York, N. Y.

*Ophthalmology*—E. Williams, M. D., Professor of Ophthalmology and Otology, Cincinnati, Ohio.

*Otology*—S. J. Jones, M. D., LL. D., Professor of Ophthalmology and Otology, Chicago, Illinois.

*Laryngology* * * *

*Dermatology and Syphilis*—A. R. Robinson, M. D., Lecturer on Dermatology and Syphilis, New York, N. Y.

*Public and International Hyhiene*—Joseph Jones, M. D., Professor of Clinical Medicine, etc., New Orleans, Louisiana.

*Collective Investigation, Vital Statistics and Climatology*—Albert L. Gihon, Medical Director U. S. Navy, Washing, D. C.

*Phychological Medicine and Nervous Diseases*—John P. Gray, M. D., LL. D., Professor of Psychological Medicine, etc., Utica, New York.

*Dental and Oral Surgery*—Jonathan Taft, M. D., Professor of Dental and Oral Surgery, Cincinnati, Ohio.

### DOMESTIC CORRESPONDENCE.

---

LIABILITY FOR MISTAKES IN DISPENSING DRUGS.

TO THE EDITORS OF THE *Chicago Medical Journal and Examiner.*

*Gentlemen :*

A recent case occurred in Iowa which has been largely noticed by medical journals within the last few weeks. The facts are that the druggist was filling a prescription, calling for extract of dandelion, from a jar containing extract of belladona, and a customer proceeded to help himself. The symptoms caused were so immediate and violent that the prescription was not dispensed. The customer brought suit against the druggist, who instituted the defense that the customer was a trespasser in helping himself, and could not hold the apothecary responsible. The defense was brushed aside by the court, who held that, if the customer took the dose from the same jar used by the apothecary, it was under an implied promise to pay, and in this view of the case he could not be held to be a trespasser. The apothecary was held liable in damages.

With the justice of the above ruling in this case but little fault can be found, but the theory of an implied promise to pay presupposes consent on the part of the druggist ; and the question then arises, what constitutes "consent," and must it be expressed or implied ? Another possible ruling, suggested by the case, is that every individual must exercise at least ordinary care to protect himself from injury or accident, and that in taking articles not directly dispensed by the druggist, he is guilty of gross carelessness and ought not to recover, though he may not be technically a trespasser.

Medicus.

